# Supplementary figures and images for: Disentangling the link between supplemental feeding, population density, and the prevalence of pathogens in urban stray cats
Source: PeerJ. 2018 Jun 25;6:e4988. doi: 10.7717/peerj.4988 (PMC6022734; doi:10.7717/peerj.4988)

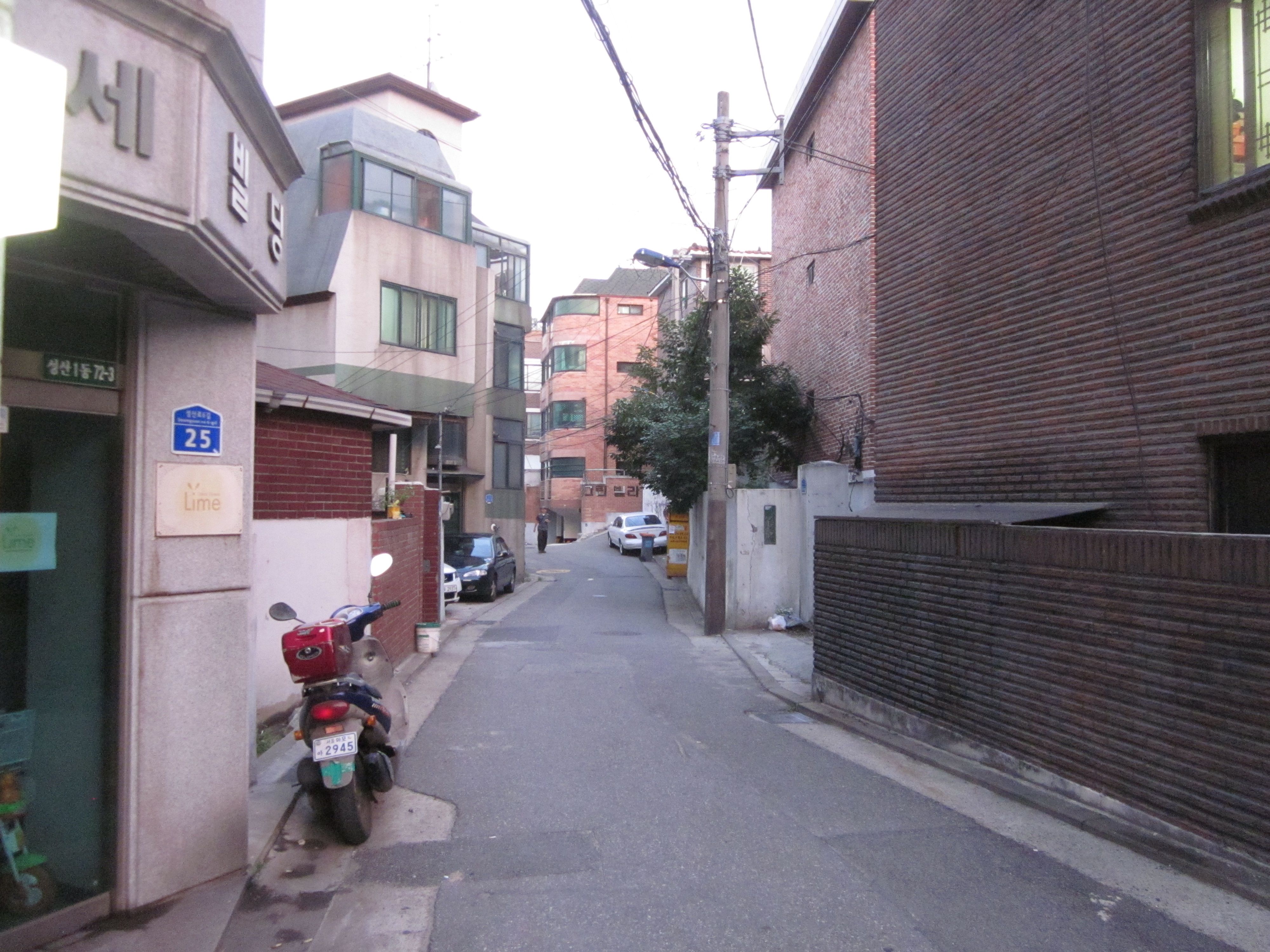

Supplement: Supplemental Information 1 — Photo by Jusun Hwang. [file peerj-06-4988-s001.jpg]
